# An open dataset with electrohysterogram records of pregnancies ending in induced and cesarean section delivery

**DOI:** 10.1038/s41597-023-02581-6

**Published:** 2023-10-02

**Authors:** Franc Jager

**Affiliations:** Faculty of Computer and Information Science, University of Ljubljana, Večna pot 113, 1000 Ljubljana, Slovenia

**Keywords:** Learning algorithms, Electromyography - EMG

## Abstract

The existing non-invasive automated preterm birth prediction methods rely on the use of uterine electrohysterogram (EHG) records coming from spontaneous preterm and term deliveries, and are indifferent to term induced and cesarean section deliveries. In order to enhance current publicly available pool of term EHG records, we developed a new EHG dataset, Induced Cesarean EHG DataSet (ICEHG DS), containing 126 30-minute EHG records, recorded early (23rd week), and/or later (31st week) during pregnancy, of those pregnancies that were expected to end in spontaneous term delivery, but ended in induced or cesarean section delivery. The records were collected at the University Medical Center Ljubljana, Ljubljana, Slovenia. The dataset includes 38 and 43, early and later, induced; 11 and 8, early and later, cesarean; and 13 and 13, early and later, induced and cesarean EHG records. This dataset enables better understanding of the underlying physiological mechanisms involved during pregnancies ending in induced and cesarean deliveries, and provides a robust and more realistic assessment of the performance of automated preterm birth prediction methods.

## Background & Summary

According to the World Health Osrganization (WHO), preterm birth, or premature birth, is defined as: Babies born alive before 37 weeks of pregnancy are completed^1^. The WHO estimates that about 15 million babies are born prematurely each year, i.e., preterm birth occurs in about 10 percent of all pregnancies. Besides medically indicated or induced preterm birth, and preterm premature rupture of membranes^2^, other pathological processes might be responsible for initiating preterm labor, such as intrauterine infection or inflammation, burst blood vessels, uterine ischemia, uterine over-distention^2^, as well as other risk factors, such as hypertension, diabetes, conization, uterine abnormalities, alcohol and drug use, smoking, and life style^3^.

Despite exhaustive research, accurate prediction of preterm birth based on these factors remains far from certain. One promising diagnostic tool for better prediction of preterm birth, weeks or even months before delivery, is a low-cost, fully- or semi-automated analysis of the uterine electromyogram, recorded from the abdominal wall of a pregnant woman, also termed as electrohysterogram (EHG). The mechanical uterine contractions present during pregnancy which are of central importance to diagnose labor are the result of discontinuous bursts of action potentials. The EHG records contain these measurable changes of the electrical potentials of the uterus thus allowing efficient non-invasive quantitative assessment of the contractions^4–10^. Applying different analysis methods showed that the EHG records contain sufficient information to diagnose labor more accurately than other traditional clinical methods^6,9,11,12^, and provide adequate data to predict preterm labor^8,9,11–14^.

The appearance of publicly available Term-Preterm EHG DataBase (TPEHG DB)^15,16^ (https://physionet.org/content/tpehgdb/) in 2011, containing 300 preterm and term spontaneous EHG records (see Table 1), recorded early (around the 23rd week) or later (around the 31st week) during pregnancy, allowed in-depth studies of non-linear signal processing techniques and machine learning approaches for accurate classification between entire preterm and term EHG records with the goal to predict preterm birth. Due to highly imbalanced sets of preterm and term EHG records (38 versus 262), researchers used a synthesis-partition over-sampling approach, based on the SMOTE^17^, or ADASYN^18^ algorithm, in order to balance the sets. Using this over-sampling approach, a number of studies using the TPEHG DB have reported near-perfect results in distinguishing between preterm and term EHG records^19–25^. In 2021, an important study^26^ revealed that over-sampling applied after data partitioning, i.e., partition-synthesis over-sampling approach, needs to be applied to achieve realistic classification performance, and realistic preterm birth prediction in the case of imbalanced sets. Recently, many interesting studies related to preterm birth prediction using the TPEHG DB were published using traditional feature engineering^27–33^ and deep learning^34–37^ approaches. A nice review of the literature dealing with the use of EHG records for the task of predicting premature birth and for understanding the underlying physiological processes during pregnancy can be found in^38^.

**Table 1:** Comparison of publicly available databases/datasets containing EHG records for EHG research, discrimination between pregnancy and labor EHG records, discrimination between preterm and term EHG records, and for predicting preterm birth.

Database/dataset	Description	Channels	Recording time	Duration	Annotations	Frequency content
TPEHG DB^15,16^	300 records,	Three EHG bipolar signals	Around the 23rd week, and around the 31st week	≈30 min	No	Bandwidth: original signals, 0–5 Hz, filtered signals, 0.08–4 Hz; *F*_S_ = 20 Hz, 16 bits
	300 pregnancies					
	- 38 preterm, 38 preg.					
	- 262 term, 262 preg.					
The Icelandic 16-electrode electrohysterogram database^16,39^	122 records,	16 EHG unipolar signals, simultaneous tocograph traces	During pregnancy (the 3rd trimester), and during labor	Pregnancy ≈61 min (19–86 min), labor ≈36 min (8–64 min)	Contractions, possible contr., movements, position changes, fetal movements, equipment manip.	Bandwidth: original signals, 0–100 Hz; *F*_S_ = 200 Hz, 16 bits
	45 pregnancies					
	- 2 preterm, 1 preg.					
	- 84 term, 30 preg.					
	- 20 induced, 7 preg.					
	- 16 cesarean, 7 preg.					
TPEHGT DS^16,23^	26 records,	Three EHG bipolar signals, simultaneous TOCO signal	Around the 31st week	≈30 min	Contraction intervals, non-contraction (dummy) intervals	Bandwidth: original signals, 0–5 Hz, filtered signals, 0.08–5 Hz; *F*_S_ = 20 Hz, 16 bits
	18 pregnancies					
	- 13 preterm, 8 preg.					
	- 13 term, 10 preg.					
	- 5 non-pregnant					
This work (ICEHG DS^62^)	126 records,	Three EHG bipolar signals	Around the 23rd week, and around the 31st week	≈30 min	No	Bandwidth: original signals, 0–5 Hz, filtered signals, 0.08–5 Hz; *F*_S_ = 20 Hz, 16 bits
	91 pregnancies					
	- 81 induced, 59 preg.					
	- 19 cesarean, 13 preg.					
	- 26 ind-ces., 19 preg.					

Another important publicly available EHG database, the Icelandic 16-electrode Electrohysterogram Database^16,39^ (https://physionet.org/content/ehgdb/) published in 2015, containing 122 EHG records (45 pregnancies), recorded during the 3rd trimester of pregnancy or during labor (see Table 1), and with human-expert annotated contractions, allowed distinguishing between pregnancy and labor. Several excellent studies have been published using this database. The studies were dedicated to discrimination^40^ and classification^41^ between pregnancy and labor groups of records, understanding human uterine electrical propagation^42^, analysis of uterine synchronization^43^, attenuation of maternal respiration signal^44^, automatic uterine contraction detection^45^ and contraction clustering^46^, recognizing uterine contractions using convolutional neural networks^47^, and to detection of preterm birth^48^.

A study dedicated to characterization of contraction and non-contraction (dummy) intervals of uterine EHG records, and automatic classification of preterm and term spontaneous EHG records^23^, yielded another publicly available dataset, i.e., Term-Preterm EHG DataSet with Tocogram (TPEHGT DS)^16,23^ (https://physionet.org/content/tpehgt/). The TPEHGT DS was published in 2018 and contains 13 preterm and 13 term spontaneous EHG records (see Table 1), recorded later (around the 31st week), with simultaneously recorded tocogram (TOCO) signal (measuring mechanical uterine activity obtained by external tocodynamometer), and with human-expert annotated contraction and non-contraction (dummy) intervals. Using this dataset, several novel approaches for classification of preterm versus term births were reported. These include the use of entire EHG records^49,50^, or individual contraction and/or dummy intervals^23,51–53^.

Unfortunately, none of these databases/dataset provide sufficient number of EHG records for reliable assessment of the accuracy of predicting imminent preterm birth. Merging EHG records from different databases/dataset may be questionable due to the differences in signal acquisition protocols. Since the EHG records of the TPEGH DB and TPEHGT DS were acquired under the same acquisition protocol, and using the same recording device, several authors merged the EHG records from these two database/dataset in cases of traditional feature engineering^24,25,32,33^ and deep learning approach^37^.

Current existing methods using uterine EHG records for predicting preterm birth solely base on classification between EHG records of which pregnancies ended in preterm spontaneous or term spontaneous delivery mode, and do not take into account other delivery modes like induced and cesarean section delivery. A robust and realistic approach for accurate prediction of preterm birth that base on the analysis of EHG records should take into account also the characteristics of EHG records of term induced and term cesarean section deliveries. Moreover, in the last 10 years the number of induced and cesarean deliveries has increased even if there is no apparent medical reason^54,55^. The latest related studies, using EHG records of induced or cesarean section delivery modes, focuses on characterization of antepartum, labor, and post-partum records^56^, characterization of bursts in late antepartum records of pregnant women with complete placenta previa^57^, differentiation between term spontaneous labor and induced late-term labor^58^, prediction of labor induction success in the first hours after induction of labor^59^, prediction of cesarean section and spontaneous vaginal delivery modes^60^, and predicting uterine atony after spontaneous or cesarean deliveries using post-partum EHG records^61^. However, these delivery-mode prediction methods relied on antepartum or labor EHG records recorded after the 37th week of gestation and did not take into account earlier recorded (before the 37th week) EHG records nor preterm birth prediction.

For these reasons, we decided to build a new EHG dataset with term induced, cesarean, and induced and cesarean EHG records, i.e., Induced Cesarean EHG DataSet (ICEHG DS)^62^, under the same acquisition protocol as was used for obtaining uterine EHG records of our previously developed TPEHG DB and TPEHGT DS. The ICEHG DS contains 126 EHG records (91 pregnancies), recorded early (around the 23rd week) and/or later (around the 31st week) during pregnancy (see Table 1), ending in induced, cesarean, or induced and cesarean delivery. Publicly available ICEHG DS^62^ will allow researchers further studies in order to answer the following important questions: (1) Can the induced and cesarean section delivery modes be predicted early, already in the 23rd, or in 31st week of pregnancy? and, (2) Can the characteristics of the EHG records of induced and cesarean section delivery modes influence the understanding of the underlying mechanisms involved during pregnancy, and more important, the understanding of the mechanisms responsible for preterm birth? Moreover, the ICEHG DS, used alongside the TPEHG DB and TPEHGT DS, will provide a robust and more realistic assessment of the performance of automated preterm birth prediction. To address some of these questions, the EHG records of the ICEHG DS, alongside the EHG records of the TPEHG DB and TPEHGT DS, have already successfully been used in one of our studies^63^. Characterization and separation of all later recorded preterm and term spontaneous, induced, cesarean, and induced and cesarean, groups of EHG records of these three database/datasets, showed that the peak amplitude of the normalized power spectra of EHG signals in the frequency band 0.125–0.575 Hz (which approximately matches the Fast Wave Low band), efficiently separate between the later preterm group and all other later term delivery groups (*p* = 2.5·10^−8^), and efficiently separate between the later preterm group and any of other later term delivery groups (*p* ≤ 4.0·10^−3^)^63^.

In summary, the areas of EHG research which have the potential to benefit from this new ICEHG DS are the following: (1) Development of efficient automated methods for pregnancy monitoring via visualization of electrical uterine activity in time and/or frequency domain; (2) Characterization and understanding physiological mechanisms involved during pregnancy that lead to induced and/or cesarean section delivery modes; (3) Development of non-invasive automated methods for prediction of induced and/or cesarean section delivery modes; (4) Mathematical modeling of electrical uterine activity; and, (5) Identification of simple and efficient EHG biomarkers for predicting pregnancy outcome. In this paper, we provide a detailed description of the ICEHG DS.

## Methods

### Data collecting

In the period from 1997 and 2006, a large number of uterine EHG records (a total of 1,211) were collected at the Clinical Department of Perinatology, University Medical Center Ljubljana, Ljubljana, Slovenia. Records were collected from the general population during routine checkups, and from the patients admitted to the hospital with the diagnosis of impending preterm labor. The records were collected either early, around the 23rd week of gestation (*early* records), and/or later in the pregnancy, around the 31st week of gestation (*later* records). The decision for the 23rd and 31st week was for the following reasons: the period from 22nd to 24th week of pregnancy (or the end of the second trimester) is an estimated border at which termination of a pregnancy is considered as an abortion, or as a delivery (i.e., extreme preterm delivery); while the 31st week of pregnancy (within the third trimester) is an estimated border after which a newborn can survive outside the uterus. (Note that these borders differ from country to country.) It was expected that characterization of the EHG records collected at these two milestones during pregnancy will provide valuable insight into changes of the physiological mechanisms involved along pregnancy. From this entire pool of uterine EHG records, in 2011 and in 2018, we developed the TPEHG DB^15,16^ (https://physionet.org/content/tpehgdb/) and TPEHT DS^16,23^ (https://physionet.org/content/tpehgt/), respectively, and made them publicly available in the Physionet repository. At these times, we were interested only in those EHG records with spontaneous preterm and spontaneous term delivery, and not in those ending in induced or cesarean section delivery. The availability of TPEHG DB and TPEHGT DS resulted in a large number of valuable studies dedicated to predicting preterm birth as outlined in Background & Summary section.

The EHG records selected for the dataset described in this paper, i.e., Induced Cesarean EHG DataSet (ICEHG DS), are also coming from the pool of EHG records collected between 1997 and 2006. Obtaining of the uterine EHG records was approved by the National Medical Ethics Committee of the Republic of Slovenia (No. 32/01/97). All women gave their written signed consent for the EHG data to be shared in a repository. The selected records for the ICEHG DS are those collected *early* and/or *later* for the pregnancies which were expected to have a normal progression toward the spontaneous start of labor and vaginal term delivery, but ultimately ended either in term vaginal delivery that failed to start spontaneously and labor had to be induced (*induced* records), in term delivery by emergency cesarean section without prior induction of labor (*cesarean* records), or in term delivery by emergency cesarean section after a failed induction (*induced-cesarean* records). The ICEHG DS is stored in the PhysioNet repository^62^.

### Recording protocol

**Fig. 1:**
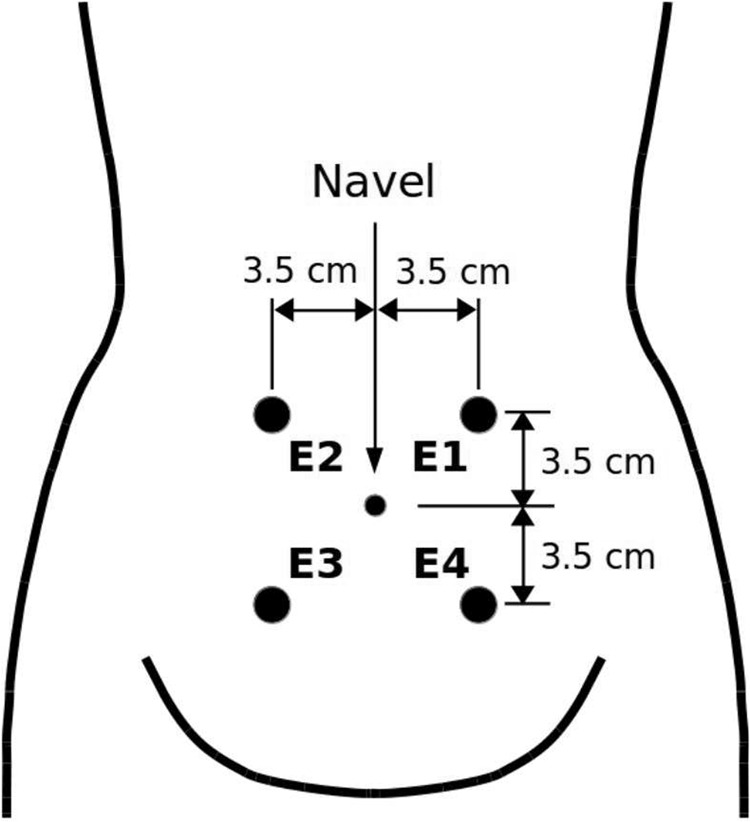
Positions of electrodes. The electrodes were placed symmetrically above and under the navel in two raws spaced at a distance of 7 cm^23^.

The recording protocol and the recording equipment were those which were also used during collecting the records of the TPEHG DB^15,16^ and TPEHGT DS^16,23^. The recording equipment consisted from a custom made physiological signal measurement device (conforming to the required ISO standards) connected to a personal computer with an integrated eight channel A/D converter. The records were collected from the abdominal surface using four Ag_2_ Cl electrodes. The electrodes were placed symmetrically above and under the navel, at the distance of 7 cm (see Fig. 1). The reference electrode was attached to the left woman’s thigh. Prior to the attachement of the electrodes, the corresponding area of 12 × 12 cm was cleaned using the acetone and ether. The precise electrode attachment positions were determined by an electrode attachement model made for this purpose. In order to lower the resistance between the electrodes, the electrode attachment positions were additionally cleaned. The four surface electrodes of the contact area of 20 mm^2^ were spread with contact conducting gel (electrode gel). In order to improve the quality of the measurements, a special protocol was used^64^. According to this protocol, the measured resistance between each pair of electrodes had to be lower than 20 kΩ. If this requirement was not reached, the electrode attachement procedure was repeated.

The acquired EHG records are of length of approximately 30 minutes and consist of three bipolar EHG signals (Fig. 1). The first acquired bipolar EHG signal was measured between the uper two electrodes, *S*1 = *E*2-*E*1, the second bipolar EHG signal between the left two electrodes, *S*2 = *E*2-*E*3, and the third bipolar EHG signal between the lower two electrodes, *S*3 = *E*4-*E*3. Prior to sampling, the signals were filtered using an analog anti-aliasing low pass three-pole Butterworth filter with the cutt-off frequency of 5.0 Hz. The sampling frequency, *F*_S_, was 20 Hz. The resolution of the signal acquisition equipment was 16 bits with the amplitude range of ±2.5 mV (A/D value of 13107 units corresponds to 1.0 mV). The sampled signals were stored on the personal computer hard disk in real time into ASCII files, while general data about the records into separate record-configuration ASCII files. No annotations of the records were provided during recording.

A record ID was assigned to each EHG record. Information on the recording time (the week of pregnancy at the event of recording) and the accompanied clinical information: age, weight, placental position, and height, were noted and stored into an .xlsx table, and added into the corresponding record-configuration ASCII file, for each participating women. Moreover, at the event of delivery, the following data were added into each record-configuration ASCII file: type of delivery (*induced*, *cesarean*, or *induced-cesarean*), gestation age, newborn weight, and ID of the pair record if EHG records were collected *early* and *later* during pregnancy.

### Data processing

**Fig. 2:**
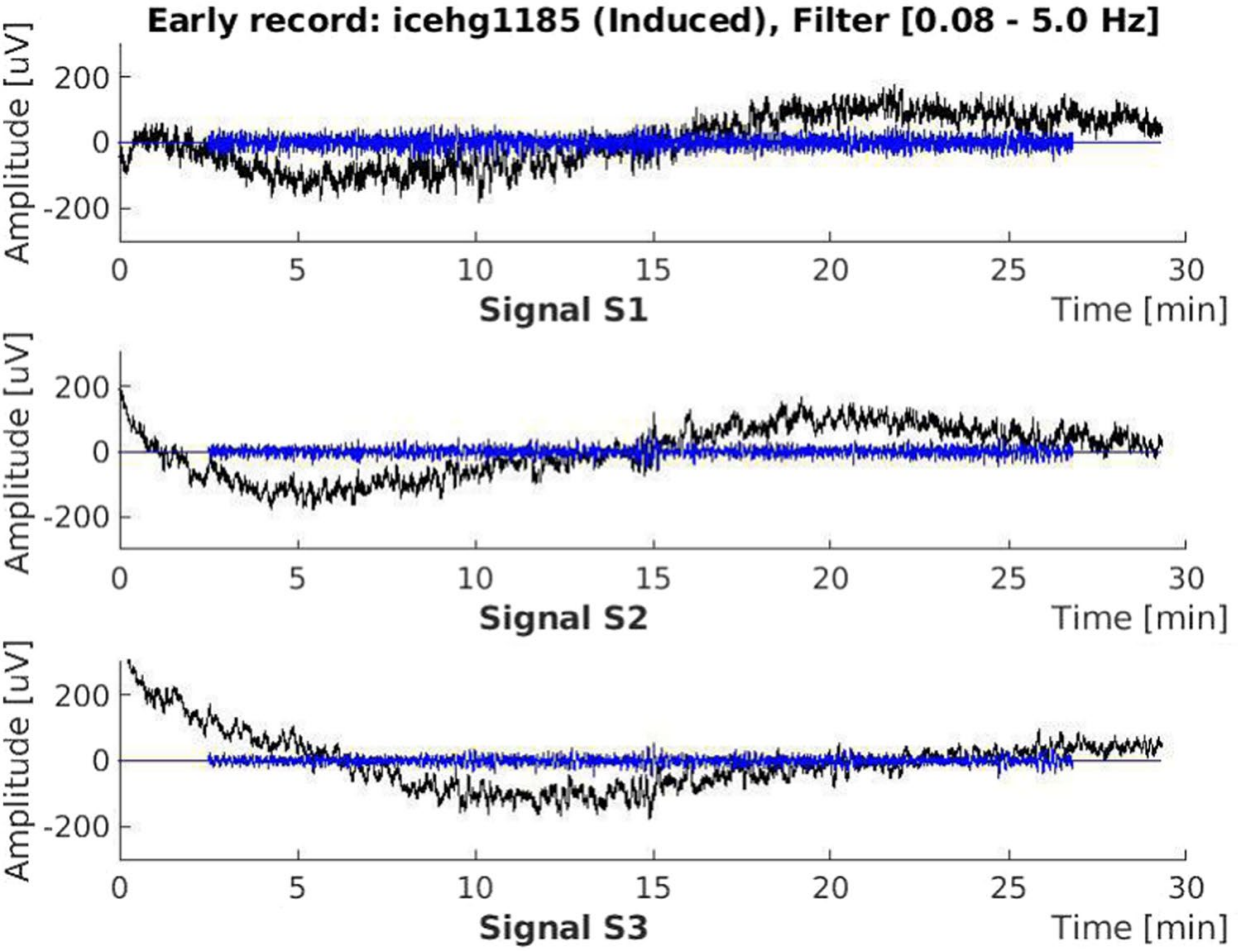
The electrohysterogram (EHG) signals of the record *icehg1185* (*induced*, delivery in the 41st week, recorded *early* in the 22nd week of pregnancy). Black: original signals, blue: filtered signals. Signal samples of the first and last 150 seconds of the filtered signals are set to zero.

In order to provide additional version of the EHG signals of the uterine EHG records obtained during recording without extremely slow signal drifts (the analog anti-aliasing filter passed frequencies from 0.0–5.0 Hz), the original EHG signals stored in the ASCII files were filtered using a four-pole digital band-pass Butterworth filter with the cut-off frequencies at 0.08 Hz and 5.0 Hz, applied bidirectionally to eliminate the non-linear phase shift. (The Butterworth filter was selected due to its nice transfer characteristic having no ripple in the pass- and stop-bands.) This processing was performed in MATLAB using readmatrix, butter, filter, flip, and plot functions. The filtered signals were then added into the original ASCII signal files of the records. An example of the EHG signals prior to and after this filtering for a selected record of the ICEHG DS is shown in Fig. 2. After that, the ASCII signal files containing original and filtered EHG signals, and the contents of the record-configuration ASCII files of the participants, were converted into the WFDB (WaveForm DataBase Software Package) record format (https://www.physionet.org/content/wfdb/) using the wrsamp WFDB application to produce binary signal (.dat) files containing very original and filtered EHG signals, and ASCII header (.hea) files. The .hea files contain information about the general data of the records, and in the comments section the accompanied clinical information of the participants. No further processing was performed in addition to this conversion. Further processing of the records is expected to be performed by the users of the dataset according to their research aims.

## Data Records

The ICEHG DS is available in the PhysioNet repository^62^. All together, the dataset contains 126 three-signal 30-minute surface EHG records coming from 91 pregnancies that were recorded *early* around the 23rd week (62 records) and *later* around the 31st week (64 records) of pregnancy. Precisely, the dataset includes 38 and 43, *early* and *later*, *induced* EHG records of 59 pregnancies (see Table 2); 11 and 8, *early* and *later*, *cesarean* EHG records of 13 pregnancies (see Table 3); and 13 and 13, *early* and *later*, *induced-cesarean* EHG records of 19 pregnancies (see Table 4). The mean times of gestation in weeks were 39.8 ± 1.4 for *induced*, 39.7 ± 1.1 for *cesarean*, and 39.4 ± 0.9 for *induced-cesarean* records.

**Table 2:** General data and accompanied clinical information (the contents of the .hea files of the EHG records of the ICEHG DS) of the participants with pregnancies ending in *induced* delivery.

Induced deliveries	Age	*Early* record	Recording time (*early*)	Weight (*early*)	*Later* record	Recording time (*later*)	Weight (*later*)	Height	Placental position	Gestation	Newborn weight
Pregnancy No.	[year]	ID	[week]	[kg]	ID	[week]	[kg]	[cm]	[front/end]	[week]	[g]
1	34	691	22.9	65				165	front	38.1	3330
2	33	713	23.1	108				164	front	39.7	4250
3	32	1064	22.7	60				160	front	41.1	3750
4	39	1077	22.3	70				163	front	39.0	3330
5	35	1086	22.1	67				165	end	38.6	2970
6	26	1148	22.6	75				174	front	40.6	4910
7	34	1235	22.7	48				154	front	39.0	2950
8	34	1248	20.9	86				164	end	37.1	2710
9	32	1297	23.4	56				173	end	39.6	3060
10	33	1303	22.7	80				161	end	41.0	3410
11	35	1394	23.4	59				160	front	41.1	3150
12	32	1408	22.3	59				159	end	37.0	2330
13	29	1454	23.1	72				162	front	40.6	3300
14	34	1496	23.6	63				163	front	38.6	3560
15	24	1568	24.1	72				165	front	39.4	2560
16	31	1570	23.6	59				162	end	39.7	3070
17	22	620	23.9	73	1529	31.9	81	168	end	41.1	3930
18	38	656	22.4	77	1385	31.1	82	167	end	41.3	3710
19	26	665	23.1	83	664	30.4	84	165	front	37.1	2860
20	32	757	22.4	68	756	30.6	72	162	end	39.7	3500
21	29	774	25.0	77	775	31.1	83	171	front	41.6	3180
22	32	806	22.4	85	805	30.1	89	158	end	41.1	3700
23	31	907	21.4	78	908	30.4	80	163	end	38.6	2360
24	28	958	22.3	79	956	30.3	82	173	end	40.0	3350
25	32	1011	22.4	70	1010	30.6	75	175	front	41.0	4380
26	35	1081	23.1	69	1079	30.9	72	157	front	37.0	2810
27	28	1124	24.4	65	1527	31.6	71	166	front	41.0	3250
28	33	1180	22.6	61	1179	30.4	64	161	front	39.6	3260
29	27	1185	22.3	62	1184	30.4	65	160	end	40.6	3590
30	33	1243	23.7	66	1238	31.6	62	166	end	40.7	3240
31	27	1361	23.1	80	989	30.9	85	170	end	40.4	3810
32	34	1388	22.4	68	1254	31.3	71	172	front	41.0	3720
33	34	1416	23.3	68	1510	30.3	69	155	end	41.3	3700
34	24	1444	22.7	74	1059	30.6	78	175	front	39.4	3020
35	29	1449	22.9	71	1040	30.9	75	163	end	38.0	3580
36	25	1599	22.1	68	668	31.0	71	175	end	39.6	2820
37	42	1680	23.9	72	865	31.1	80	169	front	39.7	3195
38	30	1698	22.3	72	799	31.3	76	170	end	41.0	3550
39	27				606	30.6	50	161	front	40.4	2710
40	32				629	31.0	63	163	front	41.1	3510
41	26				720	30.4	135	168	end	41.1	3960
42	32				784	31.7	71	160	end	38.6	2200
43	34				790	30.0	68	170	front	41.0	3190
44	27				811	31.6	67	157	end	41.7	4130
45	27				871	30.9	75	170	end	40.0	3090
46	32				924	30.9	76	178	front	41.1	3665
47	30				925	30.6	88	168	end	37.3	3560
48	40				1049	30.7	66	162	end	39.0	3385
49	29				1052	31.7	68	171	end	39.4	3320
50	29				1101	31.3	78	165	front	41.7	4450
51	31				1122	31.9	60	167	end	40.7	3300
52	21				1306	31.6	73	167	end	41.7	3400
53	25				1324	30.3	64	164	front	37.4	2820
54	30				1488	31.0	107	170	front	37.4	3550
55	32				1525	31.9	70	158	end	40.1	3480
56	32				1562	31.4	61	168	front	38.0	3450
57	35				1565	31.7	77	170	front	39.0	2540
58	28				1638	30.7	82	173	end	41.0	3270
59	24				1692	31.6	67	160	end	41.3	3140
Mean	30.7		22.9	70.7		31.0	75.2	165.5		39.8	3344
Std.	4.3		0.8	10.4		0.5	13.5	5.5		1.4	518

*Early* and *later*: recorded around the 23rd and 31st week of pregnancy.

**Table 3:** General data and accompanied clinical information (the contents of the .hea files of the EHG records of the ICEHG DS) of the participants with pregnancies ending in *cesarean* delivery.

Cesarean deliveries	Age	*Early* record	Recording time (*early*)	Weight (*early*)	*Later* record	Recording time (*later*)	Weight (*later*)	Height	Placental position	Gestation	Newborn weight
Pregnancy No.	[year]	ID	[week]	[kg]	ID	[week]	[kg]	[cm]	[front/end]	[week]	[g]
60	32	666	22.4	113				170	end	40.7	4070
61	31	976	21.7	83				180	front	40.9	4050
62	30	1089	21.9	62				160	front	38.9	2710
63	25	1187	21.9	77				166	end	39.9	4120
64	34	1443	23.0	78				173	end	38.9	3800
65	25	657	23.0	85	654	30.3	90	170	end	40.9	4110
66	27	673	24.9	75	675	30.6	79	173	front	39.1	3630
67	31	814	23.1	72	851	30.9	75	160	end	40.7	3720
68	32	885	23.3	67	714	30.7	72	168	end	40.3	4000
69	26	1175	22.1	66	1650	31.0	74	165	end	39.3	3300
70	32	1393	21.6	88	1580	30.4	93	167	front	40.6	3390
71	29				1373	31.3	91	178	end	37.1	3110
72	32				1623	31.4	70	171	front	39.0	3010
Mean	29.7		22.6	78.7		30.8	80.5	169.3		39.7	3617
Std.	3.0		1.0	14.0		0.4	9.4	6.0		1.1	473

*Early* and *later*: recorded around the 23rd and 31st week of pregnancy.

**Table 4:** General data and accompanied clinical information (the contents of the .hea files of the EHG records of the ICEHG DS) of the participants with pregnancies ending in *induced-cesarean* delivery.

Ind-cesarean deliveries	Age	*Early* record	Recording time (*early*)	Weight (*early*)	*Later* record	Recording time (*later*)	Weight (*later*)	Height	Placental position	Gestation	Newborn weight
Pregnancy No.	[year]	ID	[week]	[kg]	ID	[week]	[kg]	[cm]	[front/end]	[week]	[g]
73	28	571	23.1	61				165	front	40.4	3350
74	27	759	24.3	42				147	end	39.1	2850
75	33	959	23.7	77				159	end	38.7	3580
76	33	1091	23.7	77				171	end	40.1	3700
77	35	1131	23.7	64				165	end	41.0	3510
78	29	1682	24.0	59				162	front	40.1	3470
79	37	650	22.9	70	647	30.3	74	160	end	37.4	3300
80	33	985	22.4	65	1141	30.6	68	176	front	38.6	3110
81	27	1019	22.9	64	1430	33.9	69	171	end	38.1	2910
82	25	1053	22.4	68	1473	31.1	72	168	end	38.6	3120
83	37	1098	22.9	73	1096	30.7	76	158	front	38.9	3620
84	35	1330	23.0	58	1655	30.9	58	167	front	40.1	3390
85	32	1530	23.6	59	1218	31.7	62	162	end	39.4	3100
86	26				763	31.7	87	169	end	39.7	3660
87	24				1117	31.0	85	162	end	39.1	2780
88	28				1193	30.3	68	160	end	39.4	3980
89	30				1255	31.1	76	177	end	39.7	4140
90	25				1390	30.9	62	174	end	39.7	3430
91	27				1439	30.9	71	160	front	39.6	3540
Mean	30.1		23.3	64.4		31.2	71.4	164.9		39.4	3397
Std.	4.2		0.6	9.4		0.9	8.5	7.3		0.9	361

*Early* and *later*: recorded around the 23rd and 31st week of pregnancy.

**Fig. 3:**
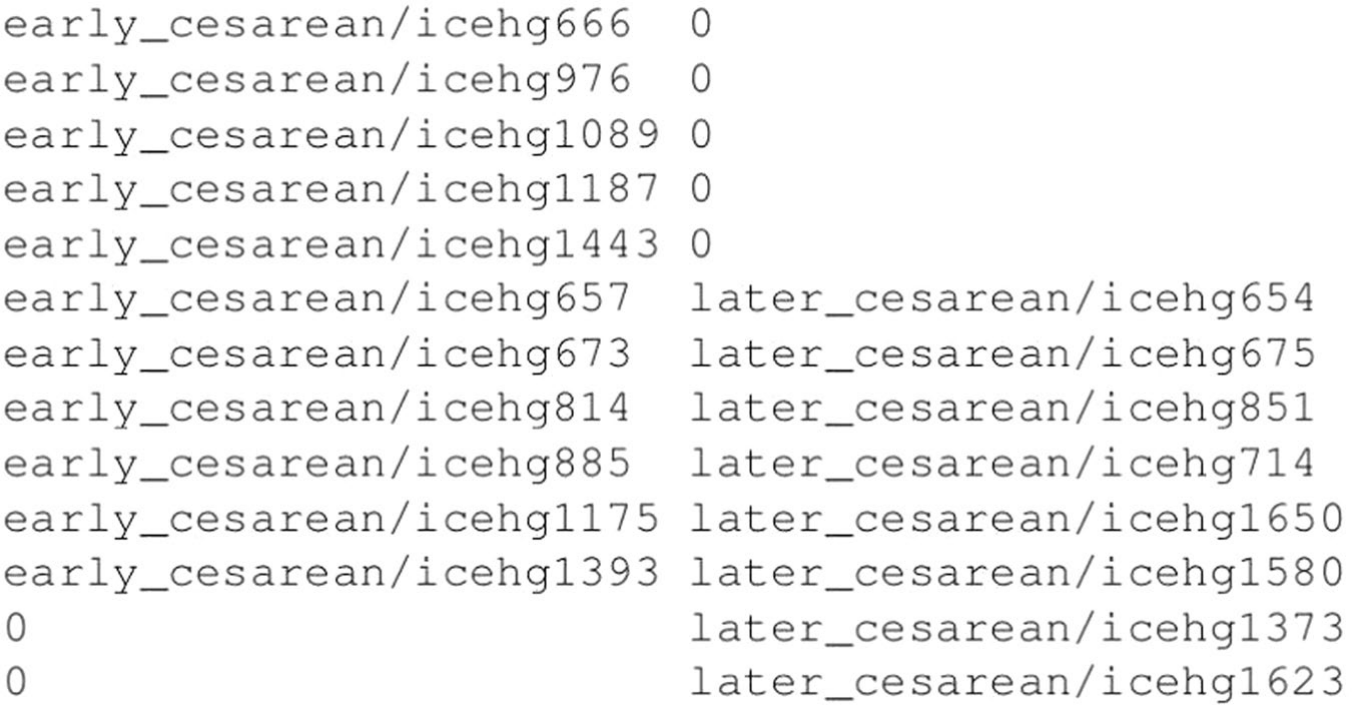
The contents of the RECORDS_cesarean file. Each raw correspons to a pregnancy ending in cesarean section and contains the name (and sub-directory name) of *early* and/or name (and sub-directory name) of *later* EHG record of the pregnancy. Columns correspond to all *early*, and all *later*, *cesarean* EHG records. A zero indicates that no *early*, or *later*, EHG record for that pregnancy exists.

Names of the records are the following: icehgXXX[X], where XXX[X] represents record ID. The entire list of records is contained in the ASCII file named RECORDS. The records are stored in the sub-directories with regard to the period of recording and according to the delivery mode, i.e., early_induced, early_cesarean, early_induced-cesarean, later_induced, later_cesarean, and later_induced-cesarean. The lists of records for each group of EHG records per delivery mode (*induced*, *cesarean*, or *induced-cesarean*) are contained in the accompanied ASCII files named RECORDS_induced, RECORDS_cesarean, and RECORDS_induced-cesarean. Each raw in these three files corresponds to a pregnancy and contains the name of *early* and/or name of *later* EHG record of the pregnancy; while if considering columns in these three files, the first column contains the names of all *early* EHG records, and the second column the names of all *later* EHG records, given the delivery mode. To better explain the contents of the latter three files, as for an example, the content of the RECORDS_cesarean file is shown and described in Fig. 3.

Each EHG record is composed from the following three files:

- A figure (icehgXXX[X]_fltrd.jpg) showing the three original EHG signals and their filtered versions (an example is shown in Fig. 2);
- a binary signal (icehgXXX[X].dat) file containing the three original EHG signals (S1, S2, and S3) and their filtered versions;
- a header (icehgXXX[X].hea) ASCII file containing the general data of the record and accompanied clinical information of the participant.

The signal data in the .dat data files are in the following order:

- original, unfiltered, signal S1;
- filtered signal S1 using a four-pole band-pass Butterworth filter from 0.08 Hz to 5.0 Hz applied bidirectionally;
- original, unfiltered, signal S2;
- filtered signal S2 using a four-pole band-pass Butterworth filter from 0.08 Hz to 5.0 Hz applied bidirectionally;
- original, unfiltered, signal S3;
- filtered signal S3 using a four-pole band-pass Butterworth filter from 0.08 Hz to 5.0 Hz applied bidirectionally.

**Fig. 4:**
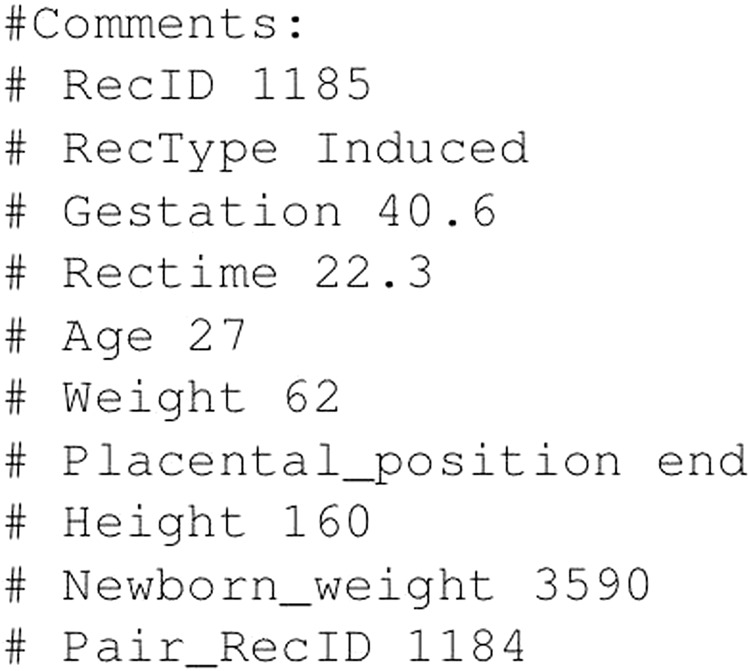
The comments section of the *icehg1185.hea* file of the record *icehg1185* (*induced*, delivery in the 41st week, recorded *early* in the 22nd week of pregnancy, the pair record, recorded *later*, is *icehg1184*).

The top most part of the .hea header files is the general data of the EHG record including: record name, sampling frequency, length of the record in samples, list of signals with their specifications according to the WFDB format, calibration constants, and signal labels. The rest of the header files is the comments section containing the accompanied clinical information of the participant. An example of the comments section of the .hea files for a selected record is shown in Fig. 4. This comments section contains the following information: record ID, type of delivery (Induced, Cesarean, or Induced-cesarean), gestation age in weeks, recording time in weeks, age of the participant in years, weight at the recording time in kg, placental position (front/end), height of the participant in cm, newborn weight in g, and ID of the pair record (if records were collected *early* and *later* during pregnancy). Tables 2–4 contain the general data and accompanied clinical information (the contents of the comments section of the .hea files) of the participants ending in *induced* (Table 2), *cesarean* (Table 3), and *induced-cesarean* (Table 4) delivery.

The records of the ICEHG DS are also available in MATLAB format (.mat signal and .hea header files) in the icehgdsmat sub-directory.

## Technical Validation

The EHG records were recorded in clinical environment. A researcher stayed with the participants throughout during recording. There were no limitations for the participants regarding talk or changing the position. The only request was not to make fast moves. The researcher continuously monitored signals and the equipment, and periodically, attachment of the electrodes. If the signal traces seemed to be very noisy (spikes, sudden step changes, signal bursts not related to contractions), all the electrode connections were verified and connections improved. During recording, the researcher did not record, nor annotate, contractions experienced by participant, fetal movements, other contraction-like electrical activities (signal bursts), noise due to movements of the participant (movement artifacts) like spikes and sudden step changes, nor other noise due to, e.g., smile or cough. Unfortunately, severe noise and artefacts appeared in some records, therefore not all EHG records were usable for the final dataset.

The EHG records of the final dataset were carefully selected from the pool of EHG records of which pregnancies ended in induced, cesarean, or induced and cesarean delivery. For the final selection of the records, the original EHG signals, and the filtered versions of the original EHG signals (using a four-pole digital band-pass Butterworth filter with the cut-off frequencies at 0.08 Hz and 5.0 Hz, applied bidirectionally), were thoroughly checked, i.e., visually inspected in time domain for the signal quality. Only those EHG records with relativly clean signals were included in the final ICEHG DS. Those EHG records showing lose of signal, extreme spikes, sudden step changes, or severe noise (bursts) of unreasonable high amplitude and duration, in their signals, or in the filtered versions of the signals, were rejected. An example of clean EHG signals (no lose of signal, no spikes, no sudden step changes, or severe noise) of a selected EHG record is shown in Fig. 2.

The technique of visual inspection of signals used in this study for selection of the final EHG records of the ICEHG DS is the same as that which was used for selecting the EHG records of our previously developed TPEHG DB and TPEHGT DS. Both, the TPEHG DB and TPEHGT DS, have already been successfuly used by many research groups, and resulted in a large number of valuable studies (see Background & Summary section). Moreover, the EHG records of the ICEHG DS have already successfully been used in one of our studies^63^. Therefore, we conclude that the technique of visual inspection of signals is reliable.

## Usage Notes

The ICEHG DS is intended to study physiological mechanisms involved during pregnancy that lead to induction, cesarean section, or both. Characterization and separation of the EHG records of the ICEHG DS can answer a question whether the induced and cesarean section delivery modes can be predicted *early*, already in the 23rd, or *later*, in the 31st week of pregnancy.

Moreover, the ICEHG DS is intended to provide more realistic pool of EHG records ending in term delivery. Since the same acquisition protocol and the same recording device were used in TPEHG DB^15,16^ (https://physionet.org/content/tpehgdb/), TPEHGT DS^16,23^ (https://physionet.org/content/tpehgt/), and in the ICEHG DS^62^, the EHG records of the ICEHG DS can be used alongside term spontaneous, *early* and *later*, EHG records of the TPEHG DB and TPEHGT DS to better understand the underlying physiologic mechanisms leading to different kinds of term delivery modes. Furthermore, adding the preterm spontaneous, *early* and *later*, EHG records of the TPEHG DB and TPEHGT DS can answer a question how the characteristics of EHG records of the induced and cesarean section delivery modes influence the understanding of the underlying mechanisms responsible for preterm birth. Such a composed pool of EHG records from all three database/datasets will provide a robust and more realistic evaluation of non-invasive automatic or semi-automatic methods for predicting preterm birth. (Note that the EHG records of these three database/datasets contain the same three bipolar signals S1, S2, and S3).

Table 5 summarizes the numbers of uterine EHG records contained in the ICEHG DS, and in our previously developed TPEHG DB and TPEHGT DS, with regard to the period of recording (*early*, *later*) and delivery mode. The percentages of EHG records in the three EHG database/datasets per delivery mode match the estimated percentages of types of deliveries in the real world only to a certain degree. According to WHO^1^, about 10 percent of all pregnancies end in preterm birth. Considering the three EHG database/datasets, they contain 11.3% of preterm spontaneous EHG records. According to NHS Maternity Statistics^65^, spontaneous delivery is the most common delivery, and has decreased from 66% to 47% in the period from 2011/12 to 2021/22. The three EHG database/datasets contain 72.1% of preterm spontaneous and term spontaneous EHG records. Furthermore, according to NHS Maternity Statistics^65^, induced deliveries has increased from 22% to 33% in the period from 2011/12 to 2021/22, and cesarean deliveries has increased from 12% to 20% in the period from 2011/12 to 2021/22. The three EHG database/datasets contain 17.9% of induced, 4.2% of cesarean, and 5.8% of induced and cesarean EHG records.

**Table 5:** The numbers of uterine EHG records contained in the TPEHG DB, TPEHT DS, and ICEHG DS.

Database/dataset	Preterm spontaneous		Term spontaneous		*Induced*		*Cesarean*		*Induced- cesarean*	
	*early*	*later*	*early*	*later*	*early*	*later*	*early*	*later*	*early*	*later*
TPEHG DB^15,16^	19	19	143	119						
TPEHGT DS^16,23^		13		13						
ICEHG DS^62^					38	43	11	8	13	13
Total (452)	19	32	143	132	38	43	11	8	13	13
Percent of Total	4.2%	7.1%	31.6%	29.2%	8.4%	9.5%	2.4%	1.8%	2.9%	2.9%
Total (452)	41		275		81		19		26	
Percent of Total	11.3%		60.8%		17.9%		4.2%		5.8%	
Total (452)	316				81		19		26	
Percent of Total	72.1%				17.9%		4.2%		5.8%	

*Early* and *later*: recorded around the 23rd and 31st week of pregnancy.

In the EHG records of the ICEHG DS three bipolar original EHG signals are stored. The first, *S*1, was measured between the upper two electrodes (see Fig. 1), the second, *S*2, between the left two electrodes, and the third, *S*3, between the lower two electrodes. Signal *S*1 and signal *S*3 estimate the uterine electrical activity in the horizontal direction, while signal *S*2 in the vertical direction. In order to better characterize the electrical activity in the vertical direction, the users of the ICEHG DS (and of the TPEHG DB and TPEHGT DS) may synthetically derive the fourth bipolar signal, *S*4, to estimate the uterine electrical activity in the vertical direction between the right two electrodes *E*4 and *E*1, *S*4 = *E*4 -* E*1. Since *S*1 = *E*2 - *E*1, *S*2 = *E*2 - *E*3, and *S*3 = *E*4 - *E*3, and using *E*4 = *S*3 + *E*3 and *E*1 = *E*2 - *S*1, it follows that:

1 $$S4=E4-E1=S3+E3-E2+S1=S1-S2+S3.$$

Similarly, bipolar signals estimating the uterine electrical activity in both diagonal directions, between the electrodes *E*2 and *E*4, and between the electrodes *E*1 and *E*3, can be derived.

The discrepancies between synthetically derived signal, *S*4, and the signal as it would be actually measured between the right two electrodes *E*4 and *E*1, are negligible. The estimated discrepancy between the calculated, *S*4, and actually measured signal, i.e., the standard deviation between the samples of these two signals (calculated throughout the 30-minute signals), is less than the difference between two adjacent integer values (0.076 *μ*V) of the signal amplitudes (1 mV/13107 = 0.076 *μ*V, where 13107 is the calibration constant relating to 1 mV), and close to the quantization error (0.038 *μ*V) of the A/D converter.

The unipolar EHG signals, as measured at the electrodes E1, E2, E3, and E4, are not stored in the EHG records, nor it is possible to synthetically derive them form the bipolar signals S1, S2, and S3.

It is good idea to set the values of signal samples of the first and last 150 seconds of the filtered version of the signals to zero (or to reject them) due to the transient effects of the Butterworth filter which was used bidirectionally for filtering the signals.

Besides the generic WFDB (WaweForm Software Package) software package (https://www.physionet.org/content/wfdb/), which was used to derive the EHG records of the ICEHG DS, the users can also use PhysioNet’s WFDB for MATLAB and Octave (https://www.physionet.org/content/wfdb-matlab/) and WFDB for Phython (https://www.physionet.org/content/wfdb-phython/) for the efficient further analysis of the records of the ICEHG DS. The records of the ICEHG DS are already readily available in MATLAB format in the icehgdsmat sub-directory. Moreover, the users can use LightWAVE, PhysioNet’s on-line signal viewer and annotation editor (https://www.physionet.org/lightwave/).

## Data Availability

During development of the ICEHG DS, the generic WFDB (WaweForm Software Package) software package (https://www.physionet.org/content/wfdb/), and WFDB for MATLAB and Octave (https://www.physionet.org/content/wfdb-matlab/), of the PhysioNet’s open-source repository were used.

## References

[1] World Health Organization, Born too soon: the global action report on preterm birth. https://apps.who.int/iris/handle/10665/44864 (accessed 16 June 2023) (2012).

[2] Goldenberg RL, Culhane JF, Iams JD, Romero R (2008). Epidemiology and causes of preterm birth. Lancet.

[3] Iams JD (2003). Prediction and early detection of preterm labor. Am Col Obstet Gynecol..

[4] Marque C, Duchene JM, Leclercq S, Panczer GS, Chaumont J (1986). Uterine EHG processing for obstetrical monitoring. IEEE Trans Biomed Eng.

[5] Devedeux D, Marque C, Mansour S, Germain G, Duchêne J (1993). Uterine electromyography: A critical review. Am. J. Obstet. Gynecol..

[6] Buhimschi C, Boyle MB, Garfield RE (1997). Electrical activity of human uterus during pregnancy as recorded from the abdominal surface. Obstet Gynecol..

[7] Leman H, Marque C, Gondry J (1999). Use of electrohysterogram signal for characterization of contractions during pregnancy. IEEE Transactions on Biomedical Engineering.

[8] Verdenik I, Pajntar M, Leskošek B (2001). Uterine electrical activity as predictor of preterm birth in women with preterm contractions. Eur J Obstet Gynecol Reprod Biol..

[9] Maner WL, Garfield RE, Maul H, Olson G, Saade G (2003). Predicting term and preterm delivery with transabdominal uterine electromyography. Obstet Gynecol..

[10] Rabotti C, Mischi M (2015). Propagation of electrical activity in uterine muscle during pregnancy: A review. Acta Physiol..

[11] Marque CK, Terrien J, Rihana S, Germain G (2007). Preterm labour detection by use of a biophysical marker: the uterine electrical activity. BMC Pregnancy Childbirth..

[12] Lučovnik M (2011). Noninvasive uterine electromyography for prediction of preterm delivery. Am J Obstet Gynecol..

[13] Maner WL, Garfield RE (2007). Identification of human term and preterm labor using artificial neural networks on uterine electromyography data. Ann Biomed Eng..

[14] Horoba K (2016). Early predicting a risk of preterm labour by analysis of antepartum electrohysterograhic signals. Biocybernetics and Biomedical Engineering..

[15] Fele-Žorž G, Kavšek G, Novak-Antolič Ž, Jager F (2008). A comparison of various linear and non-linear signal processing techniques to separate uterine EMG records of term and pre-term delivery groups. Medical & Biological Engineering & Computing.

[16] Goldberger AL (2000). PhysioBank, PhysioToolkit, and PhysioNet: components of a new research resource for complex physiologic signals. Circulation.

[17] Chawla NV, Bowyer KW, Hall LO, Kegelmeyer WP (2002). SMOTE: Synthetic Minority Over-Sampling Technique. Journal of Artificial Intelligence Research..

[18] He, H., Bai, Y., Garcia, E. A. & Li, S. ADASYN: Adaptive synthetic sampling approach for imbalanced learning. *In:**Proceedings 2008 International Joint Conference on Neural Networks (IJCNN)* 1322–1328, 10.1109/IJCNN.2008.4633969 (2008).

[19] Fergus P (2013). Prediction of Preterm Deliveries from EHG Signals Using Machine Learning. PLoS ONE..

[20] Ahmed MU, Chanwimalueang T, Thayyil S, Mandic PD (2016). A Multivariate Multiscale Fuzzy Entropy Algorithm with Application to Uterine EMG Complexity Analysis. Entropy..

[21] Fergus P, Idowu I, Hussain A, Dobbins C (2016). Advanced artificial neural network classification for detecting preterm births using EHG records. Neurocomputing..

[22] Acharya UR (2017). Automated Detection of Premature Delivery Using Empirical Mode and Wavelet Packet Decomposition Techniques with Uterine Electromyogram Signals. Comput Biol Med..

[23] Jager F, Libenšek S, Geršak K (2018). Characterization and automatic classification of preterm and term uterine records. PLOS ONE.

[24] Nieto-del-Amor F (2021). Assessment of Dispersion and Bubble Entropy Measures for Enhancing Preterm Birth Prediction Based on Electrohysterographic Signals. Sensors.

[25] Nieto-del-Amor F (2021). Optimized Feature Subset Selection Using Genetic Algorithm for Preterm Labor Prediction Based on Electrohysterography. Sensors.

[26] Vandewiele G (2021). Overly optimistic prediction results on imbalanced data: A case study of flaws and benefits when applying over-sampling. Artificial Intelligence in Medicine.

[27] Janjarasjitt S (2022). Comparison of wavelet-based decomposition and empirical mode decomposition of electrohysterogram signals for preterm birth classification. ETRI Journal.

[28] Xu J (2021). Realistic preterm prediction based on optimized synthetic sampling of EHG signal. Computers in Biology and Medicine.

[29] Far SM, Beiramvand M, Shahbakhti M, Augustyniak P (2022). Prediction of Preterm Delivery from Unbalanced EHG Database. Sensors.

[30] Lou H (2022). Bio-process inspired characterization of pregnancy evolution using entropy and its application in preterm birth detection. Biomedical Signal Processing and Control.

[31] Xu J (2022). Network Theory Based EHG Signal Analysis and its Application in Preterm Prediction. IEEE JOURNAL OF BIOMEDICAL AND HEALTH INFORMATICS.

[32] Nieto-del-Amor F (2022). Combination of Feature Selection and Resampling Methods to Predict Preterm Birth Based on Electrohysterographic Signals from Imbalance Data. Sensors.

[33] Romero-Morales H, Muñoz-Montes de Oca JN, Mora-Martínez R, Mina-Paz Y, Reyes-Lagos J (2023). J. Enhancing classification of preterm-term birth using continuous wavelet transform and entropy-based methods of electrohysterogram signals. Front. Endocrinol..

[34] Jossou TR (2022). N-Beats as an EHG Signal Forecasting Method for Labour Prediction in Full Term Pregnancy. Electronics.

[35] Rao KSN, Asha V (2023). An automatic classification approach for preterm delivery detection based on deep learning. Biomedical Signal Processing and Control.

[36] Fischer AM, Rietveld AL, Teunissen PW, Bakker PCAM, Hoogendoorn M (2023). End-to-end learning with interpretation on electrohysterography data to predict preterm birth. Computers in Biology and Medicine.

[37] Goldsztejn U, Nehorai A (2023). Predicting preterm births from electrohysterogram recordings via deep learning. PLoS ONE.

[38] Garcia-Casado J (2018). Electrohysterography in the diagnosis of preterm birth: a review. Physiological Measurement.

[39] Alexandersson A, Steingrimsdottir T, Terrien J, Marque C, Karlsson B (2015). The Icelandic 16-electrode electrohysterogram database. Sci. Data.

[40] Diab A, Boudaoud S, Karlsson B, Marque C (2021). Performance comparison of coupling-evaluation methods in discriminating between pregnancy and labor EHG signals. Computers in Biology and Medicine.

[41] Chen L, Hao Y (2017). Feature Extraction and Classification of EHG between Pregnancy and Labour Group Using Hilbert-Huang Transform and Extreme Learning Machine. Computational and Mathematical Methods in Medicine.

[42] Xu Y, Hao D, Taggart MJ, Zheng D (2022). Regional identification of information flow termination of electrohysterographic signals: Towards understanding human uterine electrical propagation. Computer Methods and Programs in Biomedicine.

[43] El Dine KB, Nader N, Khalil M, Marque C (2022). Uterine Synchronization Analysis During Pregnancy and Labor Using Graph Theory, Classification Based on Neural Network and Deep Learning. IRBM.

[44] Martins D, Batista A, Mouriño H, Russo S, Esgalhado F (2022). Palma dos Reis, C. R., Serrano, F. & Ortigueira, M. Adaptive Filtering for the Maternal Respiration Signal Attenuation in the Uterine Electromyogram. Sensors.

[45] Esgalhado F (2020). Automatic Contraction Detection Using Uterine Electromyography. Appl. Sci..

[46] Esgalhado F, Batista AG, Mouriño H, Russo S (2020). Palma dos Reis, C. R., Serrano, F., Vassilenko, V. Uterine contractions clustering based on electrohysterography. Computers in Biology and Medicine.

[47] Peng J (2019). Preliminary Study on the Efficient Electrohysterogram Segments for Recognizing Uterine Contractions with Convolutional Neural Networks. BioMed Research International.

[48] Chen L, Hao Y, Hu X (2019). Detection of preterm birth in electrohysterogram signals based on wavelet transform and stacked sparse autoencoder. PLoS ONE.

[49] Chen L, Xu H (2020). Deep neural network for semi-automatic classification of term and preterm uterine recordings. Artificial Intelligence In Medicine.

[50] Nsugbe E (2022). Novel uterine contraction signals decomposition for enhanced preterm and birth imminency prediction. Intelligent Systems with Applications.

[51] Jager F (2020). Assessing Velocity and Directionality of Uterine Electrical Activity for Preterm Birth Prediction Using EHG Surface Records. Sensors.

[52] Saleem S (2020). Granger causal analysis of electrohysterographic and tocographic recordings for classification of term vs. preterm births. Biocybernetics and biomedical engineering.

[53] Selvaraju V, Karthick PA, Swaminathan R (2023). Detection of Preterm Birth from the Noncontraction Segments of Uterine EMG using Hjorth Parameters and Support Vector Machine. Journal of Mechanics in Medicine and Biology.

[54] Antoine C, Young BK (2020). Cesarean section one hundred years 1920–2020: The good, the bad and the ugly. Journal of Perinatal Medicine.

[55] Dahlen HG (2021). Intrapartum interventions and outcomes for women and children following induction of labour at term in uncomplicated pregnancies: A 16-year population-based Linked Data Study. BMJ Open.

[56] Mas-Cabo J (2020). Robust Characterization of the Uterine Myoelectrical Activity in Different Obstetric Scenarios. Entropy.

[57] Yang J, Pan X, Garfield RE, Liu H (2020). Uterine electromyography (EMG) measurements to predict preterm caesarean section in patients with complete placenta previa. Journal of Obstetrics and Gynaecology.

[58] Alberola-Rubio J (2017). Prediction of labor onset type: Spontaneous vs induced; role of electrohysterography?. Computer Methods and Programs in Biomedicine.

[59] Benalcazar-Parra C (2019). Prediction of labor induction success from the uterine electrohysterogram. Journal of Sensors.

[60] Fergus P, Selvaraj M, Chalmers C (2018). Machine learning ensemble modelling to classify caesarean section and vaginal delivery types using Cardiotocography traces. Computers in Biology and Medicine.

[61] Diaz-Martinez A (2020). A Comparative Study of Vaginal Labor and Caesarean Section Postpartum Uterine Myoelectrical Activity. Sensors.

[62] Jager F (2023). PhysioNet.

[63] Pirnar Ž, Jager F, Geršak K (2022). Characterization and separation of preterm and term spontaneous, induced, and cesarean EHG records. Computers in Biology and Medicine.

[64] Kavšek, G. Electromiographic activity of the uterus in threatened preterm delivery. *MsC Thesis*, Faculty of Medicine, University of Ljubljana, Ljubljana (2001).

[65] NHS Digital, NHS Maternity Statistics, England - 2021-22, Chapter: Deliveries over time. https://digital.nhs.uk/data-and-information/publications/statistical/nhs-maternity-statistics/2021-22/deliveries---time-series (29 Nov 2022) (Accessed 1 July 2023).

